# Resolvin D1 and Lipoxin A_4_ Improve Alveolarization and Normalize Septal Wall Thickness in a Neonatal Murine Model of Hyperoxia-Induced Lung Injury

**DOI:** 10.1371/journal.pone.0098773

**Published:** 2014-06-03

**Authors:** Camilia R. Martin, Munir M. Zaman, Calvin Gilkey, Maria V. Salguero, Hatice Hasturk, Alpdogan Kantarci, Thomas E. Van Dyke, Steven D. Freedman

**Affiliations:** 1 Department of Neonatology, Beth Israel Deaconess Medical Center, Boston, Massachusetts, United States of America; 2 Division of Translational Research, Beth Israel Deaconess Medical Center, Boston, Massachusetts, United States of America; 3 Division of Gastroenterology, Beth Israel Deaconess Medical Center, Boston, Massachusetts, United States of America; 4 Department of Applied Oral Sciences, Center for Periodontology, Forsyth Institute, Cambridge, Massachusetts, United States of America; Vanderbilt University, United States of America

## Abstract

**Background:**

The critical fatty acids Docosahexaenoic Acid (DHA) and Arachidonic Acid (AA) decline in preterm infants within the first postnatal week and are associated with neonatal morbidities, including bronchopulmonary dysplasia (BPD). DHA and AA are precursors to downstream metabolites that terminate the inflammatory response. We hypothesized that treatment with Resolvin D1 and/or Lipoxin A_4_ would prevent lung injury in a murine model of BPD.

**Objective:**

To determine the effect of Resolvin D1 and/or Lipoxin A4 on hyperoxia-induced lung injury.

**Methods:**

C57/BL6 pups were randomized at birth to Room Air, Hyperoxia (>90% oxygen), Hyperoxia + Resolvin D1, Hyperoxia + Lipoxin A_4_, or Hyperoxia + Resolvin D1/Lipoxin A_4_. Resolvin D1 and/or Lipoxin A_4_ (2 ng/g) were given IP on days 0, 3, 6, and 9. On day 10, mice were sacrificed and lungs collected for morphometric analyses including Mean Linear Intercept (MLI), Radial Alveolar Count (RAC), and Septal Thickness (ST); RT-PCR analyses of biomarkers of lung development and inflammation; and ELISA for TGFβ_1_ and TGFβ_2_.

**Result:**

The increased ST observed with hyperoxia exposure was normalized by both Resolvin D1 and Lipoxin A_4_; while, hyperoxia-induced alveolar simplification was attenuated by Lipoxin A_4_. Relative to hyperoxia, Resolvin D1 reduced the gene expression of CXCL2 (2.9 fold), TIMP1 (6.7 fold), and PPARγ (4.8 fold). Treatment with Lipoxin A_4_ also led to a reduction of CXCL2 (2.4 fold) while selectively increasing TGFβ_2_ (2.1 fold) and Smad3 (1.58 fold).

**Conclusion:**

The histologic and biochemical changes seen in hyperoxia-induced lung injury in this murine model can be reversed by the addition of DHA and AA fatty acid downstream metabolites that terminate the inflammatory pathways and modulate growth factors. These fatty acids or their metabolites may be novel therapies to prevent or treat lung injury in preterm infants.

## Introduction

Bronchopulmonary dysplasia (BPD) is an acquired form of chronic lung disease that is unique to the preterm infant. BPD is clinically diagnosed at 36 weeks postmenstrual age or later if there is a persistent need for oxygen reflecting underlying abnormal lung development. BPD is multifactorial in its pathogenesis and, in general, is a consequence of chronic lung injury with a failure to repair and resume normal lung development. Infants with BPD experience significant clinical sequelae even after discharge from the neonatal intensive care unit including persistently altered lung function [1], [2] and poor neurocognitive outcomes in early childhood [3], [4]. Reducing the incidence of BPD would have a significant impact on quality of life as well as long term health expenditures [5]. Multiple therapies have been tested including a variety of ventilatory strategies and pharmacotherapies, but little has proved to effectively reduce BPD with some, such as postnatal steroids, resulting in potential harm.

The pathogenesis of BPD is multifactorial but is characterized by several major disease-rendering pathways: pulmonary injury, inflammation, and altered lung development, most notably alveolar simplification [6]. During fetal development the fetus is exposed to multiple biologic factors that facilitate organ development. With early delivery there is an abrupt termination in exposure to these biologic factors, many of which cannot be replaced at a rate or level that the fetus was exposed to while *in utero*. One such category of important biological factors is long chain polyunsaturated fatty acids (LCPUFAs). LCPUFAs are key mediators in organ development *in utero* through infancy and early childhood. In addition, LCPUFAs are important in regulating the inflammatory response through several mechanisms. One critical pathway is through the production of docosahexaenoic acid (DHA) and arachidonic acid (AA) derived terminal metabolites, such as Resolvin D1 and Lipoxin A_4_, respectively [7], [8].

Current options for the parenteral and enteral delivery of LCPUFAs are unable to meet estimated fetal accretion rates; and, as a result, there is a rapid deficit of DHA and AA levels with no recovery to birth levels during the neonatal course [9], [10]. Of clinical significance, this early postnatal decline in systemic DHA levels is associated with the development of BPD [10]. Animal data support this clinical observation as well as a potential role for DHA in attenuating the risk of BPD. In a neonatal murine model of hyperoxia-induced lung injury, pups exposed to hyperoxia and supplemental DHA, either by increasing the DHA content in dam milk or by direct enteral administration, demonstrated reduced lung inflammation [11] and increased alveolarization compared to pups exposed to hyperoxia without DHA [12].

However maintaining birth levels of LCPUFAs, in particular DHA and AA, is not achievable with the current standard of nutritional care in the neonatal intensive care unit [9]. Thus, in lieu of directly changing dietary DHA and AA delivery, we sought to determine whether exogenous administration of the biologically active DHA and AA derived terminal metabolites, Resolvin D1 and/or Lipoxin A_4_, would attenuate hyperoxia-induced lung injury and if so, to define the pathways modulated by these mediators.

## Methods

### Ethics Statement

All procedures were approved by the Institutional Animal Care and Use Committee at the Beth Israel Deaconess Medical Center, Boston MA (#078–2011).

### Hyperoxia-Induced Lung Injury

Breeder pairs of C57BL/6 wild type mice were housed in the Beth Israel Deaconess Animal Facility with controlled humidity and temperature environments and standard light-dark cycles. Access to food and water was available ad libitum. Dams were allowed to deliver naturally and pups were randomized within 12 hours of birth to either hyperoxia (90–95% O_2_) or room air (RA) for 10 consecutive days. The pups randomized to hyperoxia were placed in a double-walled plexiglass isolette (Air-Shields, Hatboro, PA) and a continuous flow of 11 L/min of 100% oxygen was delivered to maintain an environment of 90–95% O_2_. The dams were rotated every 24 h between the hyperoxia and RA litters to prevent oxygen toxicity. The oxygen level was monitored continuously with a MiniOX I oxygen analyzer (Ohio Medical Corporation). CO_2_ levels were monitored using a TIM10 CO_2_ analyzer (CO2meter.COM, Ormund Beach, FL). Pressure, temperature and humidity were all measured using an Atomic Clock barometer (Meade Instruments, Irvine, CA). CO_2_ levels were maintained between 426 and 1188 ppm. Humidity levels remained between 34 and 75%. The temperature inside the chamber ranged between 61 and 77° F. The barometric pressure ranged between 27.61 and 30.36 mmHg. Pup weight was recorded at birth, days 3, 6, 9, and at sacrifice. After 10 days, the pups were sacrificed by placement in a chamber of 100% CO_2_ for 10 minutes.

### Experimental Protocol

The pups were divided into five experimental groups: (1) Room Air (RA) + drug vehicle (n = 11), (2) Hyperoxia + drug vehicle (n = 12), (3) Hyperoxia + Resolvin D1 (RvD1) (n = 17), (4) Hyperoxia + Lipoxin A_4_ (LXA_4_) (n = 15), or (5) Hyperoxia + RvD1/LXA_4_ (n = 19). A total of 14 litters were used (2 RA, 2 Hyperoxia alone, 3 RvD1, 3 LXA4, and 4 RvD1/LXA4) in 10 experiments. The litter number also represents the number of experimental runs that were performed for each group. Each group had 5–6 mice per run.

RvD1 and LXA_4_ were obtained from Cayman Chemical (Ann Arbor, MI). The compounds were reconstituted in 10% ethanol (vehicle) for a final concentration of 10 ng/µl. A total of 2 ng per g pup weight of RvD1, LXA_4_, or the combination was administered IP in a volume of 50 uL on the day of randomization (day 0) and on days 3, 6, and 9 for a total of four injections. The pups in the RA or Hyperoxia alone groups were given the same volume of the drug vehicle (10% ethanol). The dose administered is within the range on 1–5 ng/gm previously reported for Resolvin analogs in adult animal models of acute lung injury [13]–[16].

On day 10, the mice were sacrificed and the lungs were removed for morphometric, quantitative RT-PCR, and ELISA analyses.

### Lung Histology and Morphometric Analyses

#### Preparation of lung tissue for histological analysis

Following euthanasia, the pups were randomly assigned for morphometric analysis or for quantitative RT-PCR analysis. For routine histology and morphometric analyses, the trachea was cannulated with a 30 gauge plastic catheter through which 10% formalin in PBS solution (Fisher Scientific) was infused. A constant perfusion pressure was maintained by suspending the burette of formalin 25 cm above the lab bench, allowing perfusion to occur by gravity over four minutes. At the end of the perfusion, the trachea was tied-off using an Ethicon surgical suture and ligated allowing for removal of the trachea and lung *in toto*. The lungs were placed in a vial containing 10% formalin in PBS solution and placed on an agitator for 1 h at room temperature followed by 4°C overnight. The lungs were washed twice with cold PBS followed by paraffin-embedding. 5 µm sections were stained with hematoxylin-eosin to examine tissue morphology and for morphometry.

#### Morphometric Analyses

Three morphometric measures, the mean linear intercept (MLI), radial alveolar count (RAC) and the mean septal wall thickness (ST), were used to determine the effect of hyperoxia on lung development and the effect of RvD1 and LXA_4_ in modulating hyperoxia-induced injury. The final number of mice per group for morphometric analyses were as follows: (1) RA + drug vehicle, n = 6; (2) Hyperoxia + drug vehicle, n = 7; (3) Hyperoxia + RvD1, n = 8; (4) Hyperoxia + LXA_4_, n = 6; and (5) Hyperoxia + RvD1/LXA_4_, n = 10. Investigators blinded to the experimental groups performed the MLI, ST (C.G.) and RAC (S.F.) measurements.

MLI represents the average alveolar diameter and was measured by photographing the slides at 100× magnification and importing these representative images into Microsoft PowerPoint. Seven evenly spaced, parallel lines composed of six 200 µm segments were placed over the image and any line that passed into a bronchiole air space, large fibrovascular vessel, or exited the lung tissue was removed. The total number of alveolar septae that crossed these lines was counted. MLI was calculated by multiplying the number of 200 µm segments used by the length of the segments (200 µm) and then dividing that number by the number of alveolar septum intercepts [17], [18]. The data are expressed as mean +/− SEM.

RAC quantifies alveolar septation and is a measure of alveologenesis. Using images at 100× magnification, a perpendicular line was drawn from a respiratory bronchiole to the nearest pleural edge or fibrovascular septum. Airspaces or saccules that traversed this line were counted [17], [19]–[21]. The data are expressed as mean +/− SEM.

Changes in septal thickness (ST) represent alterations in the extracellular matrix and/or cellular components within this matrix. The mean ST was calculated by using images photographed at 200× magnification. Images were then imported into Microsoft PowerPoint and 5 parallel, equidistant lines were placed across the image. At the point where the alveolus crossed the horizontal line, the width of the septal wall was measured along its perpendicular plane [22]. The data are expressed as mean +/− SEM.

### Quantitative real-time RT-PCR

Real time quantitative reverse transcriptase-PCR (RT-PCR) (Qiagen, SABiosciences) was used to quantify the relative changes in gene expression induced by hyperoxia and modified by RvD1 and/or LXA_4_ treatment during hyperoxia. The final number of mice per group for RT-PCR analyses were as follows: (1) RA + drug vehicle, n = 5; (2) Hyperoxia + drug vehicle, n = 5; (3) Hyperoxia + RvD1, n = 9; (4) Hyperoxia + LXA_4_, n = 9; and (5) Hyperoxia + RvD1/LXA_4_, n = 9.

Genes were grouped into four physiologic categories of lung development and injury - Cell Differentiation & Organogenesis; Growth Factor Signaling; Extracellular Matrix, and Inflammation (Table 1).

**Table 1:** Biomarkers assayed by RT-PCR in lung tissue.

Cell Differentiation & Organogenesis	Growth Factor Signaling	Extracellular Matrix	Inflammation
TGFβ_1_, TGFβ_2_	BMPR1B and 2	Eln	CRP
TGFβreceptor II	Smad1-5	Col1a1	IL-1β
VEGF-A		LOXL2	CD46
PPARα, γ		MMP-2, -9	ICAM-1
		TIMP1	CCL5
			TNF-α
			CXCL2

TGF, transforming growth factor; VEGF, vascular endothelial growth factor, PPAR, peroxisome proliferator-activated receptor; BMP, bone morphogenetic protein; Eln, Elastin; Col1a1, Collagen, Type 1, alpha 1; TIMP1, TIMP metallopeptidase inhibitor 1; LOXL2, lysyl oxidase homolog 2; MMP, matrix metalloproteinase; CRP, C-reactive protein; IL, interleukin; CXCL2, chemokine (C-X-C motif) ligand 2; CD46, CD46 complement regulatory protein; ICAM-1, intracellular adhesion molecule 1; CCL5, Chemokine (C-C motif) ligand 5; TNF, tumor necrosis factor.

At sacrifice, the lung tissues were snap frozen using liquid nitrogen and stored at −80°C. Total RNA was isolated using RNeasy lipid tissue mini kit (Qiagen, Frederick, MD) following manufacturer instructions. This consisted of taking approximately 50 mg of snap-frozen lung tissue that was then disrupted and homogenized in 1 ml QIAzol lysis reagent using a Tissueruptor (Qiagen, Frederick, MD). The samples were incubated at room temperature for 5 min. 200 µl chloroform was added to each sample, shaken vigorously for 15 seconds and incubated at room temperature for 3 min. The samples were centrifuged at 12,000×g for 15 min at 4°C. The upper aqueous phase was transferred to a new tube and in an equal volume of 70% ethanol was added and vortexed. The sample was transferred to an RNeasy column placed in a 2 ml tube. Extraction was followed by a DNase digestion step to remove any contaminating genomic DNA by adding 10 µl of DNase I to an RNeasy column membrane. Then the column was washed with RW1 and RPE buffer. Finally the RNeasy column was placed in new 1.5 ml tube, 30 µl RNase-free water was added, centrifuged for 1 min at 8000×g, and flow-through was collected. RNA concentration and purity was measured using a Nanodrop 2000 (Thermo Scientific). cDNA was synthesized with RT^2^ First Strand kit (Qiagen, Frederick, MD) using 2 µg total RNA, diluted with 91 µl of nuclease-Free water and stored in −80°C for further analysis.

An RT^2^ rofiler Custom PCR Array was used to simultaneously examine the mRNA levels of 27 genes, the housekeeping gene GAPDH, and three controls in 96-well plates according to the protocol of the manufacturer (SA Biosciences, Frederick, MD). RT-PCR was performed on an ABI Prism 7700 Sequence Detector (Applied Biosystems, Foster City, California) using a standard cycling protocol. The total cycle number was 40. Ct values were determined and mRNA expression levels were calculated using the Δ-Δ Ct-method with expression levels of the respective mRNAs normalized to GAPDH.

### TGFβ2 and TGFβ1 ELISA

TGFβ2 and TGFβ1 were quantified using a Quantikine ELISA Kit from R&D Systems following the manufacturer's instructions. Sample acidification was performed to obtain immunoreactive TGFβ by incubating the samples with 1 N HCl for 10 minutes, and neutralized using 1.2 N NaOH/0.5 M HEPES. 25 µg of protein was used for the TGFβ2 assay, whereas 1.7 µg of protein was used for the TGFβ1 assay. Recombinant mouse TGFβ2 and TGFβ1 were used as the standard.

### Statistical Analysis

Pup weights and lung morphometric measures were expressed as means ± SEM. One-way ANOVA correcting for multiple comparisons was used to compare pup growth, lung morphometric measures, and TGFβ2 and TGFβ1 protein levels across experimental groups.

RT-PCR results were summarized and displayed as median fold change (± IQR) where a value above one represents up-regulation from control values and a value below one represents down-regulation compared to control values. The 2∧-ΔCt values for each gene of interest were used to determine statistically significant differences in gene expression between groups. The Kruskal–Wallis test was performed to evaluate differences in gene expression between the experimental groups. If the result of the Kruskal-Wallis test was statistically significant, then Wilcoxon rank-sum tests were used to compare the groups in a pairwise fashion [23].

All analyses were performed using STATA statistical software, version 13 (StataCorp) and GraphPad Prism version 6.00 for Windows, GraphPad Software (San Diego, CA, www.graphpad.com).

## Results

### Neonatal Pup Growth

**Figure 1:**
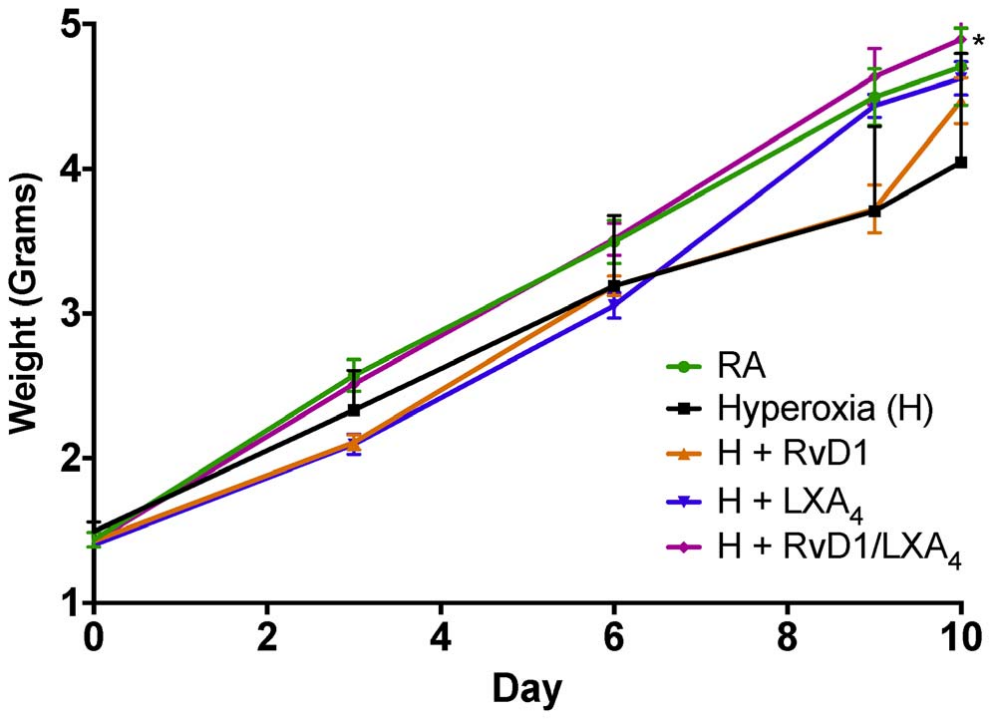
Pup growth patterns by experimental group. RA, room air; H, hyperoxia; RvD1, Resolvin D1; LXA_4_, Lipoxin A_4_. *p<0.05 compared to Hyperoxia group.

The mean pup weight in the RA group on the day of allocation (within 12–24 hours of birth, day 0) was 1.44 ± 0.05 grams (Figure 1). The range in day 0 weights for the hyperoxia and treatment groups ranged from 1.40 to 1.49 grams. There were no statistical differences between the experimental groups at day 0.

On day 10, the mean pup weight in the RA group was 4.71 ± 0.27 grams. The range in day 10 weights for the remaining groups was 4.05 to 4.90. The final weights were statistical different between the experimental groups (p = 0.04), with the significant difference being between the RA and Hyperoxia alone groups (p = 0.04) and between the Hyperoxia alone and Hyperoxia + RvD1/LXA_4_ group (p = 0.003). In addition to absolute weight gain, growth velocity (grams of weight gain per day relative to their birth weight, gm/d) was also determined. Overall growth velocity was different across the groups (p = 0.01). Significant pairwise comparisons were obtained between the RA and Hyperoxia alone groups, 0.23 ± 0.03 versus 0.17 ± 0.01 gm/d (p = 0.009); and between Hyperoxia alone and the Hyperoxia + LXA_4_ and Hyperoxia + RvD1/LXA_4_ groups at 0.23 ± 0.01 gm/d (p = 0.008) and 0.24 ± 0.01 gm/d (p = 0.008), respectively.

### Lung Histology and Morphometric Analyses

**Figure 2:**
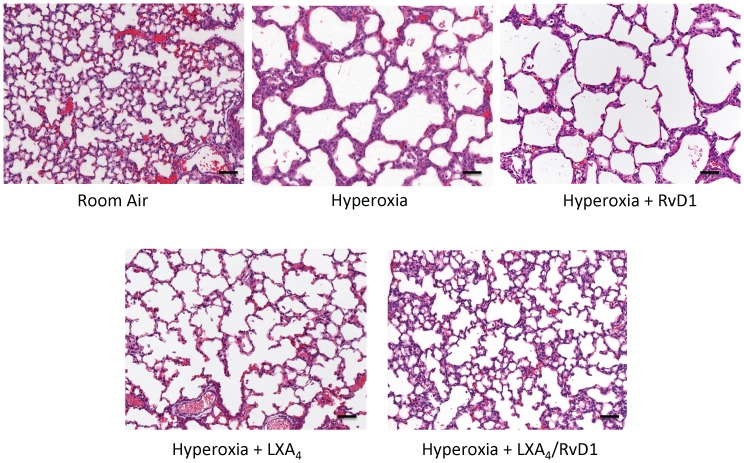
Lung histology by treatment group. Representative images of H and E stained sections were taken at an original magnification of 200x. Bars represent 100 µm. RvD1, Resolvin D1; LXA_4_, Lipoxin A_4_.

Representative lung histology images taken at 200× magnification are shown in Figure 2. In the RA group, well-formed terminal alveoli were seen with no inflammatory infiltrates or septal wall thickening. In contrast, hyperoxia-exposed mice demonstrated marked alveolar dilation referred to as alveolar simplification in conjunction with lumenal inflammatory infiltrates. The septal walls were noticeably thicker. Treatment with RvD1 resulted in attenuation of septal wall thickening but no improvement in alveolar simplification. LXA_4_ exposure resulted in similar results to RvD1 but additionally was associated with partial improvement in alveolar simplification. The combination of RvD1/LXA_4_ reduced alveolar simplification and septal wall thickening approximating morphological characteristics seen in RA mice.

**Figure 3:**
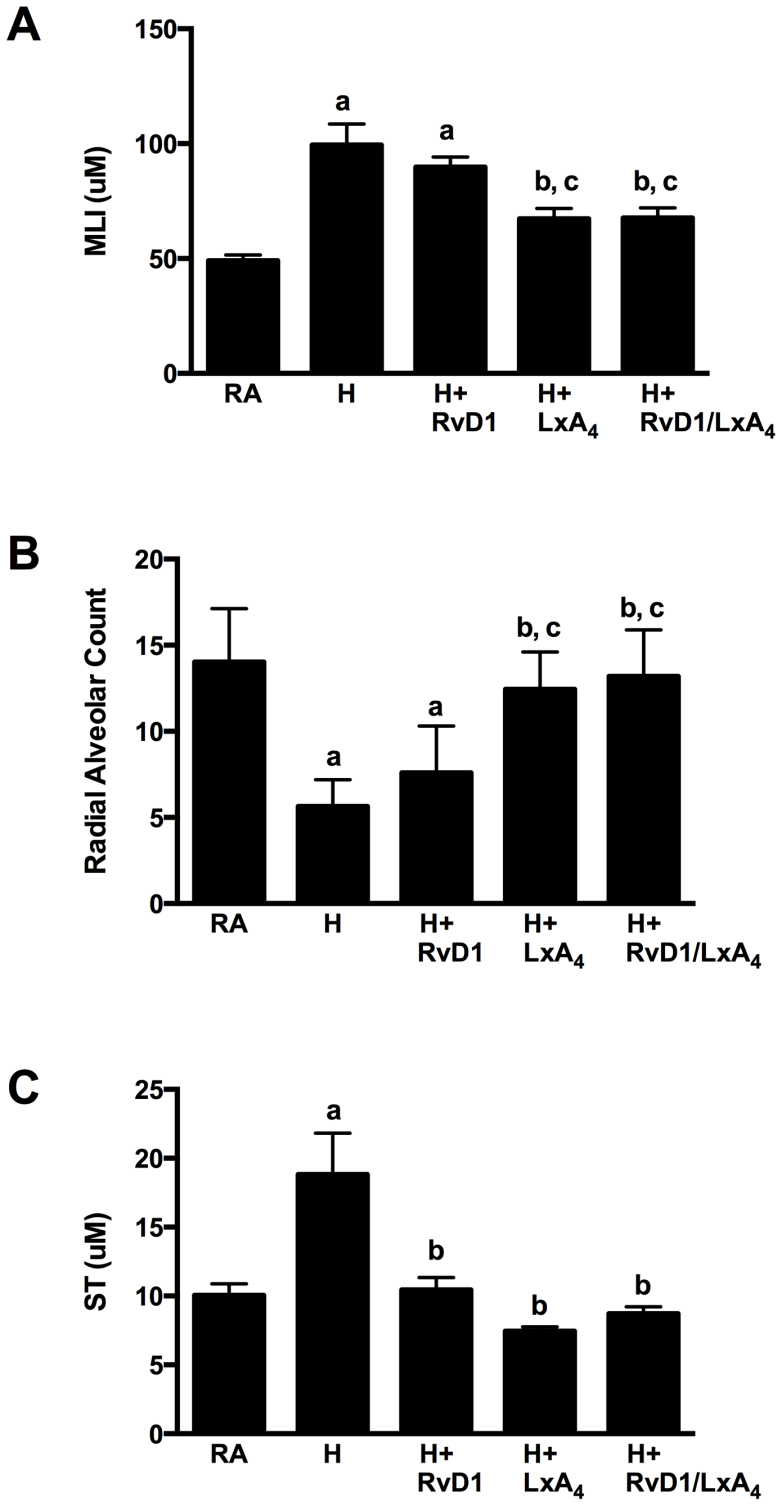
Morphometric assessment of lung histology. Shown in Figure 3A, B, and C are the morphometric assessment of the mean linear intercept (MLI), radial alveolar count (RAC) and septal thickness (ST) respectively. Bars represent means +/- SEM. 37 mice were analyzed for the MLI and ST morphometric measures. A mean of 6 images were analyzed per section/mouse. 34 mice were analyzed for RAC. A mean of 3 images were analyzed per section per mouse. RA, room air; H, hyperoxia; RvD1, Resolvin D1; LXA_4_, Lipoxin A_4_ a = p<0.05 compared to RA group; b = p<0.05 compared to Hyperoxia alone group; c = p<0.05 compared to the Hyperoxia + RvD1 group.

To quantitate the differences between the different experimental groups, well-established morphometric analyses were performed using MLI, RAC and ST (Figure 3). Hyperoxia-induced lung injury increased the MLI from a mean of 49.0 ± 2.5 µm in the RA group to 99.4 ± 9.2 µm (p<0.0001, Figure 3A). Compared to hyperoxia, there was no change in the MLI with RvD1 treatment (89.8 ± 4.5 µm). In contrast, treatment with LXA_4_ and the combination of RvD1/LXA_4_ significantly decreased the MLI to 67.3 ± 4.5 µm (p = 0.006) and 67.7 ± 4.4 µm (p = 0.002), respectively.

The Hyperoxia alone group demonstrated a decrease in the RAC values compared to the RA group with a mean count of 5.6 ± 0.6 from 14.0 ± 1.3 in the RA group (p<0.0001, Figure 3B). The Hyperoxia + RvD1 group did not significantly change this count compared to the Hyperoxia alone group. In contrast, both Hyperoxia + LXA_4_ and Hyperoxia + RvD1/LXA_4_ increased the RAC relative to Hyperoxia alone (12.4 ± 0.9, p = <0.0001; 13.2 ± 0.9, p = <0.0001; respectively) with counts approaching those observed in the RA group. The RAC increases in the latter two groups were also significantly different when compared to the Hyperoxia + RvD1 group (p = 0.002 and p<0.0001, respectively).

Compared to RA, the mean ST increased from 10.0 ± 0.8 µm to 18.8 ± 3.0 µm (p = 0.003) with hyperoxia (Figure 3C). Compared to hyperoxia, all three treatment groups significantly decreased the mean ST: RvD1 (10.4 ± 0.9 µm, p = 0.002), LXA_4_ (7.5 ± 0.3 µm, p<0.0001), and the combination RvD1/LXA_4_ (8.7 ± 0.5 µm, p<0.0001).

### Quantitative real-time RT-PCR (Table 2): Changes in gene expression with hyperoxia exposure relative to room air

**Table 2:** Fold change in gene expression within studied physiologic categories of lung development across experimental groups.

	Experimental Groups Median Fold Change (IQR)							
Physiologic Category	Hyperoxia^a^		H+ RvD1^b^		H+ LXA_4_ b		H+ RvD1/LXA_4_ b	
Cell Differentiation & Organogenesis	Fold Change	(IQR)	Fold Change	IQR	Fold Change	IQR	Fold Change	IQR
TGFβ_1_	0.62	(0.76)	0.58	(0.16)	0.94	(0.78)	0.66	(0.22)
TGFβ_2_	**0.31** **	(0.06)	1.45	(1.15)	**2.14** *	(2.47)	**2.55** **	(1.02)
TGFBRII	0.74	(0.66)	1.55	(1.34)	1.33	(0.67)	1.72	(1.07)
VEGF-A	0.37	(0.80)	0.84	(0.27)	1.44	(1.02)	0.97	(0.58)
PPARα	0.64	(0.98)	1.55	(1.26)	1.17	(0.76)	1.01	(0.60)
PPARγ	1.13	(0.63)	**0.21**	(0.45)	0.85	(0.83)	**0.28** **	(0.12)
**Growth Factor Signaling**								
BMPR1B	0.66	(0.39)	0.69	(0.32)	1.07	(0.78)	0.67	(0.34)
BMPR2	**0.22**	(0.17)	0.55	(0.31)	1.27	(0.69)	0.92	(0.63)
Smad1	**0.44** *	(0.04)	0.78	(0.48)	1.11	(0.33)	1.00	(0.54)
Smad2	0.54	(0.31)	0.58	(0.28)	0.76	(1.31)	0.57	(0.17)
Smad3	**0.40** *	(0.14)	1.51	(0.40)	**1.58** **	(0.56)	1.59	(0.71)
Smad4	**0.47** **	(0.25)	0.82	(0.35)	1.43	(1.13)	1.05	(0.43)
Smad5	**0.34**	(0.07)	0.67	(0.48)	0.82	(0.29)	0.66	(0.47)
**Extracellular Matrix**								
Eln	**0.19**	(0.28)	1.63	(1.23)	0.95	(1.34)	0.82	(0.29)
Col1a1	**0.60** *	(0.31)	1.41	(1.25)	1.07	(0.49)	1.23	(1.04)
LOXL2	**0.42** **	(0.15)	1.52	(0.55)	1.36	(0.51)	1.26	(0.63)
MMP-2	**0.25**	(0.08)	1.29	(0.71)	1.17	(0.27)	1.08	(0.55)
MMP-9	0.74	(0.70)	0.65	(0.59)	0.88	(0.36)	0.67	(0.24)
TIMP1	**9.96** **	(42.26)	**0.15** *	(0.32)	0.69	(0.52)	**0.22**	(0.33)
**Inflammation**								
CRP	1.44	(3.37)	0.86	(3.98)	0.51	(1.20)	0.81	(0.95)
IL-1β	1.67	(2.93)	0.57	(0.75)	1.12	(0.53)	1.06	(1.12)
CD46	0.56	(0.53)	**0.28**	(0.29)	1.00	(0.78)	**0.39**	(0.27)
ICAM-1	0.86	(0.27)	0.83	(0.39)	**1.55** *	(0.60)	1.16	(0.23)
CCL5	**0.33**	(0.15)	**0.45**	(0.77)	0.82	(0.45)	0.50	(0.33)
TNF-α	0.85	(0.20)	**2.07**	(0.98)	**2.68**	(2.10)	**3.08** **	(1.10)
CXCL2	**3.72** **	(107.28)	**0.35**	(0.11)	**0.41**	(0.85)	**0.49**	(0.42)

**Bold**  =  greater than a 2-fold change and/or p<0.05; values <1 represent down-regulation;

values >1 represent up-regulation.

a Relative to the Room Air group;

b Relative to the Hyperoxia alone group.

*p = 0.05,

**p<0.05.

#### Biomarkers of cell differentiation and organogenesis

Hyperoxia resulted in a greater than two fold decrease in the gene expression of the growth factors VEGF-A and TGFβ_2_, with the latter showing statistical significance (p = 0.009). There was also reduced expression of TGFβ_1_ and PPARα, but this was less than a 2-fold change and was not statistically significant.

#### Biomarkers of growth factor signaling

BMPR2, Smad1, Smad3, Smad4, and Smad 5 all demonstrated a decrease in gene expression by 2-fold change or greater with exposure to hyperoxia. Of these changes, Smad1 (p = 0.047), Smad3 (p = 0.047), and Smad4 (p = 0.009) were statistically different when compared to the gene expression of these factors in the RA group.

#### Biomarkers of the extracellular matrix

Compared to the RA group, exposure to hyperoxia increased the gene expression of the profibrogenic protein TIMP1 (p = 0.03). Gene expression for Eln, LOXL2, and MMP-2 demonstrated a greater than 2-fold negative change, with only LOXL2 reaching statistical significance (p = 0.009). Col1A1 also demonstrated a decrease in expression, yet this change was less than 2-fold although was statistically significant (p = 0.047).

#### Biomarkers of inflammation

Relative to RA, hyperoxia demonstrated a 2-fold or greater increase in the gene expression only for CXCL2, the murine equivalent of IL-8, (p = 0.02). Although there were increases in IL-1β and CRP, the relative change compared to the RA group was less than 2 fold. Only CCL5 demonstrated a greater than 2-fold decrease in response to hyperoxia, however this was not statistically different. CD46 demonstrated a small, but less than 2-fold, decrease in expression with hyperoxia exposure. The other factors analyzed did not appreciably change including ICAM-1 and TNF-α.

### Modulation in the Gene Expression of Hyperoxia-Induced Biomarkers of lung injury by RvD1 and LXA_4_

#### Biomarkers of cell differentiation and organogenesis

LXA_4_ and the combination of RvD1/LXA_4_ increased TGFβ_2_ by greater than 2-fold compared to the level of gene expression induced by hyperoxia; this change was statistically different (p = 0.02) with pairwise significance reached for both treatment groups (p = 0.05 and p = 0.009, respectively). Additionally, RvD1 and RvD1/LXA_4_ decreased PPARγ by greater than 2-fold compared to hyperoxia; this change was statistically different (p = 0.02) with pairwise significance reached for the combination group alone compared to the hyperoxia group (0.009). No other genes showed a 2 fold or greater change.

#### Biomarkers of growth factor signaling

All of the three treatment groups, RvD1, LXA_4_ and RvD1/LXA_4_, increased the gene expression of Smad3, but this change was less than 2-fold, although the pairwise comparison between Hyperoxia alone and Hyperoxia + LXA4 was significant (p = 0.04). The remaining signaling proteins evaluated did not appreciably change in the three treatment groups compared to levels induced by hyperoxia.

#### Biomarkers of the extracellular matrix

RvD1 and the combination of RvD1/LXA_4_ led to a greater than 2-fold decrease in the gene expression of TIMP1 compared to hyperoxia alone. The reduction in TIMP1 for RvD1 was at a significance level of 0.05. No other genes showed a 2 fold or greater change.

#### Biomarkers of inflammation

Relative to hyperoxia alone, RvD1, LXA_4_, and the combination of RvD1/LXA_4_ in the presence of hyperoxia, led to a 2-fold change or greater in the gene expression of only CXCL2, although none of these changes reached statistical significance. RvD1 additionally decreased CCL5, and CD46. LXA_4_ alone did not negatively influence the gene expression of these factors, however, it did increase the expression of ICAM-1 (p = 0.05). All three treatment groups increased the gene expression of TNF-α by greater than 2-fold (p = 0.04), with the significant pairwise comparison being between the hyperoxia and the combination group (p = 0.003). None of the three treatment groups significantly affected the gene expression of CRP or IL-1β.

#### TGFβ2 and TGFβ1 ELISA

**Figure 4:**
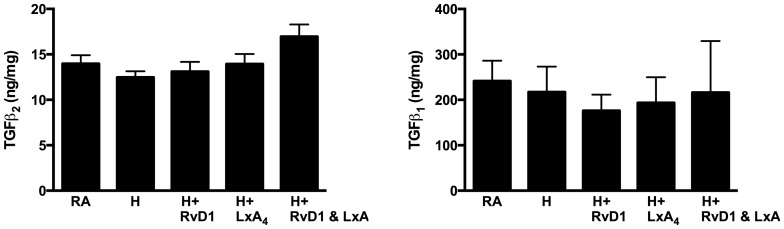
TGFβ_2_ and TGFβ_1_ ELISA. RA, room air; H, hyperoxia; RvD1, Resolvin D1; LXA_4_, Lipoxin A_4_.

To evaluate further the RT-PCR changes found in TGFβ2 gene expression across the experimental groups, we performed an ELISA for TGFβ2. We also examined TGFβ1 to determine if the relative values seen in RT-PCR for both TGFβ2 and TGFβ1 parallel the changes in protein expression. The combined Hyperoxia + RvD1/LXA4 group had increased TGFβ2 protein levels (17.0 ± 1.3 ng/mg) compared to Hyperoxia alone (12.5 ± 0.7 ng/mg, p = 0.007) and Hyperoxia + RvD1 (13.1 ± 1.1 ng/mg, p = 0.02) (Figure 4). However, the overall ANOVA across all five experimental groups was short of statistical significance with a p-value of 0.08. For TGFβ1, the Hyperoxia + RvD1 group had decreased TGFβ1 protein levels (176.2 ± 12.5 ng/mg) compared to the RA group (217.0 ± 18.7 ng/mg, p = 0.04), however, the overall ANOVA across all groups was not significant (p = 0.3). These results demonstrate that the changes seen in mRNA expression in TGFβ2 and TGFβ1 parallel the relative changes seen in protein expression across the experimental groups.

## Discussion

In a well-established neonatal model of lung injury, the administration of the bioactive terminal metabolites of DHA and AA, RvD1 and LXA_4_ respectively, attenuated the morphologic and cellular responses to hyperoxia-induced lung injury. In parallel, there was improvement in pup growth with the combination of RvD1/LXA_4_, which was principally driven by LXA_4_. These findings support a mechanistic role for fatty acid derived terminal metabolites in ameliorating specific pathways that contribute to severe lung disease in preterm infants. In addition, these results may explain the association of low systemic levels of DHA to an increased risk of BPD observed in clinical studies [10].

### Hyperoxia-induced lung injury

Consistent with previous studies, we found that exposure of mice to hyperoxia in the early neonatal period disrupts normal lung development as evidenced by the morphometric changes of increased septal wall thickness and arrested alveologenesis [24], [25]. Also, consistent with previous studies, is the induction of the host inflammatory response with hyperoxia exposure [26]–[28]. In our study, we demonstrated an increase in the gene expression of CXCL2, the murine equivalent of IL-8, and to a lesser extent IL-1β? We did find in parallel an increase in the gene expression of TIMP1 with a concomitant decrease in the expression of ELN, LOXL2, and Col1A1, specific factors that contribute to the structure and integrity within the extracellular matrix [29]–[31]. The presence of inflammation and disorganization of the extracellular matrix are key elements in the development of BPD which are observed in both animal models as well as human infant studies [32]. Furthermore, the fact that hyperoxia exposure led to a decrease in VEGF-A, a critical factor for angiogenesis, concurs with the current understanding that this protein is critical for normal alveoli development [33], [34].

Our finding of a significant decrease in TGFβ_2_ with hyperoxia exposure as well as the other signaling proteins important in the TGF-BMP-Smad pathway is in contrast to some studies of neonatal hyperoxia-induced lung injury where an elevation of TGF-β isoforms are seen with hyperoxia exposure [35]–[37]. There are a couple of possible explanations for this discrepancy. First, many studies measured only TGFβ_1_, the predominant isoform, and although we saw a lowering in gene expression with hyperoxia, it was not statistically different from the control or normoxia group. Our hyperoxia model is of 10-day duration and longer durations of hyperoxia exposure may be needed to see a change in TGFβ_1_ [38]. It is possible that there is a biphasic change in TGFβ isoforms similar to VEGF where a decrease is observed early in the course of disease evolution and this is followed by a later increase. Little data exists to the timing and changes seen in TGFβ_2_ gene expression with hyperoxia and its specific role in lung development. However, our finding of decreased expression of TGFβ_2_ and Smad 3 with hyperoxia and concomitantly alveolar simplification and, in contrast, an increase in TGFβ_2_ and Smad 3 in the LXA_4_ and combination groups with parallel improved alveolarization is consistent with scientific literature examining the complex role of TGFβ isoforms in lung development. This duality of TGFβ function in lung development under different conditions is supported by Vincencio et al. demonstrating that TGFβ can induce changes of BPD when over expressed between postnatal days P7 and P14 in a murine model [18]. Yet in contrast, knockout mouse models of Smad3 overlapping with this time period indicate that the TGFβ/Smad3 signaling pathway can beneficially induce alveolarization [39]. Thus the timing of the change in TGFβ and Smad3 expression relative to the period in lung development may be the defining factor as to whether it inhibits or promotes airway maturation.

### Effects of the fatty acid terminal metabolites, RvD1 and LXA_4_, in the attenuation of hyperoxia-induced lung injury

Long chain polyunsaturated fatty acids, including DHA and AA, can modulate the inflammatory response by inhibiting NFκB activation, through PPARγ induction and/or by direct synthesis of bioactive metabolites that have an active role in terminating inflammation [40]. RvD1 and LXA_4_ are biosynthesized from the precursors DHA and AA, respectively, through sequential lipoxygenase steps [41]. In adult animal models of lung disease, both RvD1 and LXA_4_ have demonstrated potent anti-inflammatory effects [42]. However, although some aspects may be shared in the pathophysiology of adult and neonatal lung injury, the neonate, especially the preterm infant, is unique in that lung development is still ongoing and the compromise in normal developmental processes also contributes to the specific features in neonatal lung injury.

Our date indicate that RvD1 impacted the expression of genes for both inflammation (CXCL2) and the extracellular matrix biomarker (TIMP1), both of which may have contributed to reducing septal wall thickness. RvD1 did not have a substantial effect alone on VEGF-A or the other growth factor biomarkers in the TGFβ family. Nor did RvD1 impact the expression of the BMP-Smad signaling proteins. This is consistent with the morphological results where no change in MLI or RAC, thus alveologenesis, was observed from what was quantified with exposure to hyperoxia. The actions of RvD1 in our model of hyperoxia-induced lung injury is consistent with results by Rogers et al., whereby increasing pup exposure to DHA via supplemented dams (and thus dam milk) led to improvement in inflammation but no change in the characteristic alveolar simplification seen with hyperoxia [11].

Similar to RvD1, LXA_4_ demonstrated a reduction in septal wall thickening with values similar to that of the lungs in healthy, normoxia mice. However, unique to LXA_4_ was the improvement in alveogenesis with a reduction in MLI and increase in RAC approaching those seen in the Room Air group. The selective change in gene expression induced by LXA_4_ that may account for this morphometric difference between the two groups is the increase in expression of TGFβ_2_ and to a lesser degree, the increase in Smad3. It is possible that this change in TGFβ_2_ at the gene and protein level is unrelated to the improvement of alveologenesis observed with LXA_4._ However, TGFβ has been described to have an important role across the spectrum of alveolar development including preservation of normal alveologenesis, as described above [39]. Whether an increase at the protein level of Smad3 occurs with LXA_4_ needs to be studied further to corroborate the trends observed in gene expression.

In summary, this study identified several candidate pathways by which fatty acid derived terminal metabolites may attenuate hyperoxia-induced lung injury. These pathways include their well-established role in regulating inflammation, but also include novel pathways in modulating the extracellular matrix and activating TGFβ_2_-Smad3. Additional interrogation of these pathways, across different doses and including identification of the cell types involved in these pathways, are important next steps. In addition, it is important to note that in adult models of acute lung injury other pathways of attenuation have been identified with the use of these agents emphasizing their pleiotropic effects [13]–[16], [43].

The current study has several important implications. This is the first paper to demonstrate the role and potential pathways by which long chain polyunsaturated fatty acid derived terminal metabolites ameliorate a common neonatal morbidity that is characterized by both dysregulated inflammation and altered organogenesis. Second, it begins to offer biologic plausibility to the clinical studies documenting a relationship between systemic DHA levels and the risk of BPD. Lastly, this is the first description of the role of RvD1 and LXA_4_ in modulating neonatal organogenesis. The deficits in systemic levels DHA and AA, as observed in the early postnatal period in the preterm infant, potentially would also lead to lower availability of RvD1 and LXA_4_. Thus, strategies to replete DHA and AA during the early postnatal period of the preterm infant and/or administration of RvD1 and LXA_4_ may represent potential therapeutic strategies to ameliorate the development of BPD.
